# The sensitivity to change of the cluster headache quality of life scale assessed before and after deep brain stimulation of the ventral tegmental area

**DOI:** 10.1186/s10194-021-01251-5

**Published:** 2021-06-06

**Authors:** Davide Cappon, Agata Ryterska, Harith Akram, Susie Lagrata, Sanjay Cheema, Jonathan Hyam, Ludvic Zrinzo, Manjit Matharu, Marjan Jahanshahi

**Affiliations:** 1 Unit of Functional Neurosurgery, Department of Clinical and Movement Neurosciences, University College London (UCL) Institute of Neurology, National Hospital for Neurology and Neurosurgery, 33 Queen Square, WC1N 3BG London, UK; 2 Hinda and Arthur Marcus Institute for Aging Research, Hebrew SeniorLife, Harvard Medical School, Boston, MA USA; 3 Deanna and Sidney Wolk Center for Memory Health, Hebrew SeniorLife, Boston, MA USA; 4 Department of Psychology, Queen Mary University of London, London, UK; 5 Headache and Facial Pain Group, UCL Queen Square Institute of Neurology, The National Hospital for Neurology and Neurosurgery, London, UK; 6 MOE Key Lab for Neuroinformation, The Clinical Hospital of Chengdu Brain Science Institute, University of Electronic Science and Technology of China, Chengdu, China

**Keywords:** Cluster headache, Trigeminal autonomic cephalalgias, Quality of life scale, Deep brain stimulation

## Abstract

**Background:**

Cluster headache (CH) is a trigeminal autonomic cephalalgia (TAC) characterized by a highly disabling headache that negatively impacts quality of life and causes limitations in daily functioning as well as social functioning and family life. Since specific measures to assess the quality of life (QoL) in TACs are lacking, we recently developed and validated the cluster headache quality of life scale (CH-QoL). The sensitivity of CH-QoL to change after a medical intervention has not been evaluated yet.

**Methods:**

This study aimed to test the sensitivity to change of the CH-QoL in CH. Specifically we aimed to (i) assess the sensitivity of CH-QoL to change before and following deep brain stimulation of the ventral tegmental area (VTA-DBS), (ii) evaluate the relationship of changes on CH-QoL with changes in other generic measures of quality of life, as well as indices of mood and pain. Ten consecutive CH patients completed the CH-QoL and underwent neuropsychological assessment before and after VTA-DBS. The patients were evaluated on headache frequency, severity, and load (HAL) as well as on tests of generic quality of life (Short Form-36 (SF-36)), mood (Beck Depression Inventory, Hospital Anxiety and Depression Rating Scale), and pain (McGill Pain Questionnaire, Headache Impact Test, Pain Behaviour Checklist).

**Results:**

The CH-QoL total score was significantly reduced after compared to before VTA-DBS. Changes in the CH-QoL total score correlated significantly and negatively with changes in HAL, the SF-36, and positively and significantly with depression and the evaluative domain on the McGill Pain Questionnaire.

**Conclusions:**

Our findings demonstrate that changes after VTA-DBS in CH-QoL total scores are associated with the reduction of frequency, duration, and severity of headache attacks after surgery. Moreover, post VTA-DBS improvement in CH-QoL scores is associated with an amelioration in quality of life assessed with generic measures, a reduction of depressive symptoms, and evaluative pain experience after VTA-DBS. These results support the sensitivity to change of the CH-QoL and further demonstrate the validity and applicability of CH-QoL as a disease specific measure of quality of life for CH.

## Introduction

Quality of life (QoL) scales have increasingly emerged as an  essential clinical outcome measure for assessing the impact of a disorder, the symptoms, and its medical or surgical treatment on patients’ well-being and daily life. Trigeminal autonomic cephalalgias (TACs) are a group of primary headaches including cluster headache (CH), paroxysmal hemicrania, hemicrania continua, and short-lasting unilateral neuralgiform headache attacks [1]. CH is the most common form of TAC and is characterized by a highly disabling headache that is strictly unilateral (with occasional side switching). CH causes excruciating pain associated with prominent cranial autonomic features and a sense of restlessness or agitation. Quality of life studies in patients with CH have shown limitations in normal daily functioning as well as in social functioning and family life [2, 3].

Specific measures to assess QoL are lacking and assessment of quality of life in this population is currently limited to the use of a combination of tests including generic quality of life scales such as the SF-36 [4]. However, these measures might not be specifically sensitive for CH and might, for example, fail to discriminate between CH patients and migraineurs, highlighting the need for a specific scale to assess QoL in CH [5].

In light of this, we previously developed and validated (on a total of 406 patients) the first patient-reported outcome measure to specifically monitor QoL in patients with CH in clinical care and research [6]. It was shown that the cluster headache quality of life scale (CH-QoL) has essential psychometric properties, including good construct validity, convergent validity, internal consistency and test retest reliability [6]. An important aspect of the validity of a clinical scale is its sensitivity to change, most importantly reflecting change after a medical or surgical intervention [7].

In a significant number of highly disabled individuals, standard medical therapy is not sufficiently effective to treat headache attacks in CH. For these patients, ventral tegmental area deep brain stimulation (VTA-DBS) has been demonstrated to reduce the frequency, severity and duration of headache attacks, and to lower anxiety levels and pain seeking behavior associated with the attacks [8, 9]. DBS is a surgical treatment in which electric pulses are continuously applied via stereotactically implanted electrodes and is now considered as a therapeutic option for refractory CH with proven efficacy [10].

The aim of the present study was to evaluate the sensitivity to change of the CH-QoL. Specifically, the aims were (i) to assess the sensitivity of CH-QoL to change before and after VTA-DBS intervention, (ii) to assess the association of change on CH-QoL with change in other generic standardized measures of quality of life, as well as indices of mood and pain in CH.

## Methods

### Study population

Ten consecutive patients with cluster headache undergoing VTA-DBS at the National Hospital for Neurology and Neurosurgery in London UK were enrolled (Table 1). All enrolled participants underwent clinical examination, neuropsychological assessment and completed the CH-QoL prior to surgery and one year or longer post-operatively. The surgical procedure has been described previously [8] and involved DBS lead (model 3389, Medtronic Inc.) implantation in the ipsilateral VTA or bilaterally (if symptoms were side alternating) under local or general anesthesia.

**Table 1:** Demographic and headache characteristics. M = male, F = female, HAL = headache load, DBS = deep brain stimulation, M = mean, SD = standard deviation. Age, education and duration units are years.

ID	Gender	Age	Education	Duration	Side of attacks	Headache frequency pre-DBS	Headache frequency post-DBS	Headache severity pre-DBS	Headache severity post-DBS	HAL pre-DBS	HAL post-DBS
1	M	46	11	7	Right	3–4/day	3/day	6–8/10	5–6/10	275	156
2	F	41	19	4	Bilateral	3–5/day	1/day	6–9/10	5–6/10	1964	347
3	M	58	10	14	Left	2/day	1–2/day	6–8/10	7–9/10	840	520
4	F	42	17	21	Bilateral	2–4/day	2–7/day	9–10/10	9–10/10	2178	2543
5	M	43	10	28	Right	5–15/day	8/day	7–8/10	9–10/10	865	994
6	M	37	11	23	Right	5/day	4–5/week	9–10/10	8–9/10	764	78
7	M	39	11	13	Bilateral	3–6/day	12/month	5–9/10	5–7/10	700	20
8	M	41	16	15	Left	7–10/day	2–3/day	8–10/10	6–7/10	1387	75
9	M	59	11	20	Left	5–7/day	6–7/day	7–9/10	7–8/10	519	603
10	M	48	11	15	Right	3/day	1–2/day	2–6/10	2–6/10	379	198
	80 % M	M 45.4 SD (11.9)	12.7 (2.9)	16.0 (7.1)						1964 (649)	347 (762)

### Assessment of headache frequency, severity and load

Data on headache frequency, duration and severity were obtained from a “headache diary” completed by patients at the relevant time points. Headache severity was evaluated on a verbal rating scale (VRS) for pain (0, no pain, to 10, the worst pain imaginable). Headache frequency was described as the number of CH episodes per day. Headache load (HAL) is a composite score to simultaneously measure frequency, severity and duration of cluster headache episodes. It was calculated as Σ (severity [verbal rating scale] x duration [in hours]) of all headache attacks experienced over a 2-week period [8].

### Cluster headache quality of life scale (CH-QoL)

CH-QoL scale consists of 28 items that are answered on a four points scale (Never = 0 to Always = 4). In addition to a total score, 4 sub-scores factors can be derived: F1 Restrictions of activities of daily living items 1–9; F2 Impact on mood and interpersonal relationships items 10–21; F3 Pain and anxiety items 22–23; F4 Lack of vitality items 24–28. The total scores range from 0 to 112 with *higher* scores indicating *poorer* health related quality of life [6] (seeTable 2).

**Table 2:** Cluster headache quality of life scale (CH-QoL) total and subscale scores before (pre-op) and after ventral tegmental area deep brain stimulation (VTA-DBS)

ID	F1 pre-op	F1 post-VTA DBS	F2 pre-op	F2 post-VTA DBS	F3 pre-op	F3 post-VTA DBS	F4 pre-op	F4 post-VTA DBS	CH-QoL TOT pre-op	CH-QoL TOT post-VTA DBS
1	22	10	8	7	5	5	12	12	47	34
2	27	12	22	14	6	1	16	10	71	37
3	27	24	20	13	4	4	15	16	66	57
4	36	36	31	22	8	8	18	17	93	83
5	35	33	30	32	7	7	14	16	86	88
6	35	26	30	36	4	6	18	15	87	83
7	31	29	36	32	7	8	18	16	92	85
8	30	33	37	33	7	7	14	16	88	89
9	27	30	19	29	6	8	17	20	69	87
10	24	16	32	26	8	8	13	10	77	60
M	29.4	24.9	26.5	24.4	6.2	6.2	15.5	14.8	77.6	70.3
(SD)	(4.8)	(9.2)	(9.1)	(9.9)	(1.5)	(2.3)	(2.2)	(3.2)	(14.5)	(21.6)

F1 restriction of activities of daily living, F2 impact on mood and interpersonal relationship, F3 pain and anxiety, F4 lack of vitality, *M* mean, *SD* standard deviation, *TOT* total score, *VTA-DBS* ventral tegmental area deep brain stimulation

### Assessment of generic quality of life, mood and pain

#### Generic quality of life scale -SF-36

The *Short Form-36* (SF-36) [11] is a 36-item questionnaire which measures generic Quality of Life (QoL) across eight domains (physical and social functioning, physical and emotional role limitations, mental health, energy, pain, and general health perceptions). Eight different sub-scores, and a physical and mental summary score, can be derived. The maximum score ranges from 0 (lowest or worst possible level of functioning) to 100 (indicates the best possible health state).

### Mood

The *Beck Depression Inventory* (BDI-II) [12] is a self-report measure of the severity of depression with regard to cognitive, affective, somatic, or behavioral symptoms. Scores range from 0 to 63, with higher scores denoting higher depression.

The *Hospital Anxiety and Depression Rating Scale* (HADS) [13] is a self-report measure assessing depression and anxiety. The sum of items in each subscale represents a total score indicating global anxiety (HADS-A) or depression (HADS-D). On both Depression and Anxiety subscales scores range from 0 to 21, with higher scores indicating more severe depression or anxiety.

### Pain

The *Headache Impact Test* (HIT-6) [14] is a six-item questionnaire used to measure the adverse impact of headaches on role and social functioning, cognitive functioning, vitality,psychological distress, and pain severity. The scores range from 36 to 78, and functional impact due to headaches can then be categorized into four groups: little or no impact (< 49), some impact (50–55), substantial impact (56–59), and severe impact (60–78).

The *McGill Pain Questionnaire* (MPQ) [15] is a measure of subjective pain experience that includes 78 adjectives describing the quality of pain, divided across four domains, namely sensory, affective, evaluative, and miscellaneous aspects of pain. The total possible score ranges from 0 to 78, with higher scores indicating worse pain.

*The Pain Behaviour Checklist* (PBC) [16] is a self-report assessment to quantify three classes of pain behaviours: help seeking, avoidance, and complaint.

### Data analysis

All data were analyzed using the computing environment R [17]. Means and standard deviations were calculated for all variables. Paired samples t-tests were used to examine whether a significant change in CH-QoL, SF-36 domains, mood and/or pain had occurred from before to after VTA-DBS. Pearson correlational analyses were performed to explore the relationship between the change scores in the CH-QoL scale (before and after VTA-DBS) and change scores in measures of the SF-36, mood, and pain. We calculated and reported 95 % confidence intervals for all analyses.

Cohen’s d effect size was calculated for the CH-QoL total score and its 4 subdomains:

$$Cohe{n}^{`}s d = \frac{mean(post)-mean(baseline)}{Standard Deviation } $$

A d value of 0.2–0.4 reflects a small effect, 0.5–0.7 an intermediate effect, and 0.8 -1 a large effect.

Standardized response mean (SRM) was calculated for the CH-QoL total score and the foursubdomains:

$$SRM= \frac{mean(post)-mean(baseline)}{Standard Deviation {(\Delta)} } $$

Cohen’s d and SRM are standardized indices of power to detect a true change, and larger values indicate higher sensitivity to change [7, 18].

## Results

There was some individual variability in the effects of VTA-DBS on HAL (see Tables 1 and 3). While the majority of 7 patients showed significant and clinically notable improvement of their HAL after VTA-DBS compared to before surgery, this was not the case for patients 4, 5 and 9.

### CH-QoL scale sensitivity to change with VTA-DBS

**Fig. 1:**
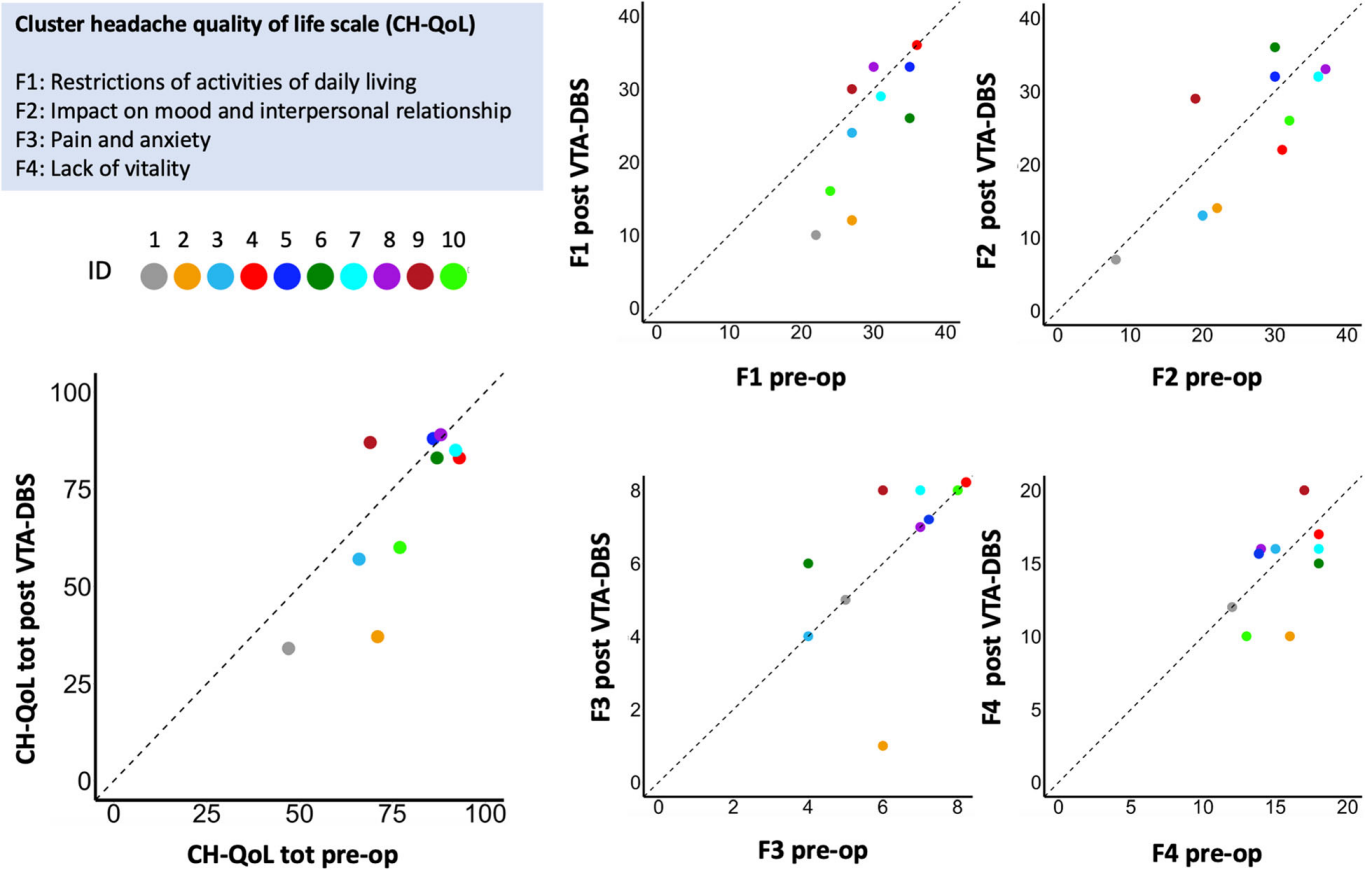
Scatter plots depicting changes in CH-QoL scale total score and 4 sub-scores. Diagonal line represents x equal to y or before equal to after VTA-DBS, points that fall under the diagonal line depict a reduction in CH-QoL scale score after VTA-DBS treatment. Note: Lower scores on CH-QoL indicate better health related quality of life. CH-QoL cluster headache quality of life scale, DBS deep brain stimulation, F factors of the cluster headache quality of life scale

Participants were evaluated within one month prior to the DBS procedure and one year or more post-operatively (mean 12 months SD = 1.8). CH-QoL total score and the four sub-domains scores for pre and post VTA are presented in Table 3; Fig. 1. The CH-QoL total score was significantly reduced after (M = 70.3, SD = 21.6) compared to before VTA-DBS (M = 77.6, SD = 14.5), t(9) -2.0, *p* = 0.03, *d* = -0.6), indicating better health-related quality of life reported by the patients after VTA-DBS. The CH-QoL ‘restriction of daily activities’ score was also significantly reduced after (M = 24.90, SD = 9.29) compared to before VTA-DBS (M = 29.40, SD = 4.8), t(9) -2.28, *p* = 0.001, *d* = -0.7), indicating better daily functioning after surgery. The scores on the other two subscales ‘mood and interpersonal relationships’ and ‘lack of vitality’ subscales were lower after (respectively M = 24.40, SD = 9.9; M = 14.80, SD = 3.19) compared to before VTA-DBS (respectively M = 26.50, SD = 9.1; M = 15.50, SD = 2.2), indicating better health-related functioning of patients after VTA-DBS. However, the change on these subscales was not significant (see Tables 3 and Fig. 1). For the CH-QoL total score and the CH-QoL restriction ADL Cohen’s d were respectively 0.6 and for 0.7, indicating intermediate effects. These results suggest that the CH-QoL scale is sensitive to change and particularly the total score and the activities of daily living subscore are significantly improved following improvement of headaches after VTA-DBS surgery.

**Table 3:** CH-QoL sensitivity to change for the patients with cluster headache who underwent ventral tegmental area deep brain stimulation (VTA-DBS).

CH-QoL subscale	Baseline (SD)	Post (SD)	Mean Diff	Paired t-test (95 % CI)	*p*-values	Cohen’s d	SRM
ADL Restriction	29.4 (4.8)	24.9 (9.2)	-4.5	-2.28 (-6.8, -0.2)	0.01*	-0.7	-0.7
Mood & interpersonal relations	26.5 (9.1)	24.4 (9.9)	-2.1	-1.52 (-5.4, 0.9)	0.14	-0.3	-0.4
Pain and anxiety	6.2 (1.5)	6.2 (2.3)	0.0	-1.02 (-1.4, 0.47)	0.30	-0.3	-0.3
Lack of vitality	15.5 (2.2)	14.8 (3.2)	-0.7	-1.08 (-2.6,0.9)	0.29	-0.3	-0.2
CH-QoL total score	77.6 (14.5)	70.3 (21.6)	-7.3	− 2.05 (− 14.4,0.3)	0.03*	-0.6	-0.5
**Clinical Outcome**							
HAL^+^	987.1 (649.5)	553.45 (761.7)	-443.7	-2.60 (-858.5, -102)	0.01∗	-0.6	-0.6

ADL activities of daily living, CH-QoL cluster headache quality of life scale, CI confidence interval, HAL headache load (composite of frequency, severity and duration of cluster headache episodes), Mean DIFF = Mean Post – Mean Baseline, SD standard deviation, *SRM *standard mean response,* significant *p*-values ^+^ cluster headache patients

### Correlation of CH-QoL scale total change score (before and after VTA-DBS) with change scores of the other measures

**Fig. 2:**
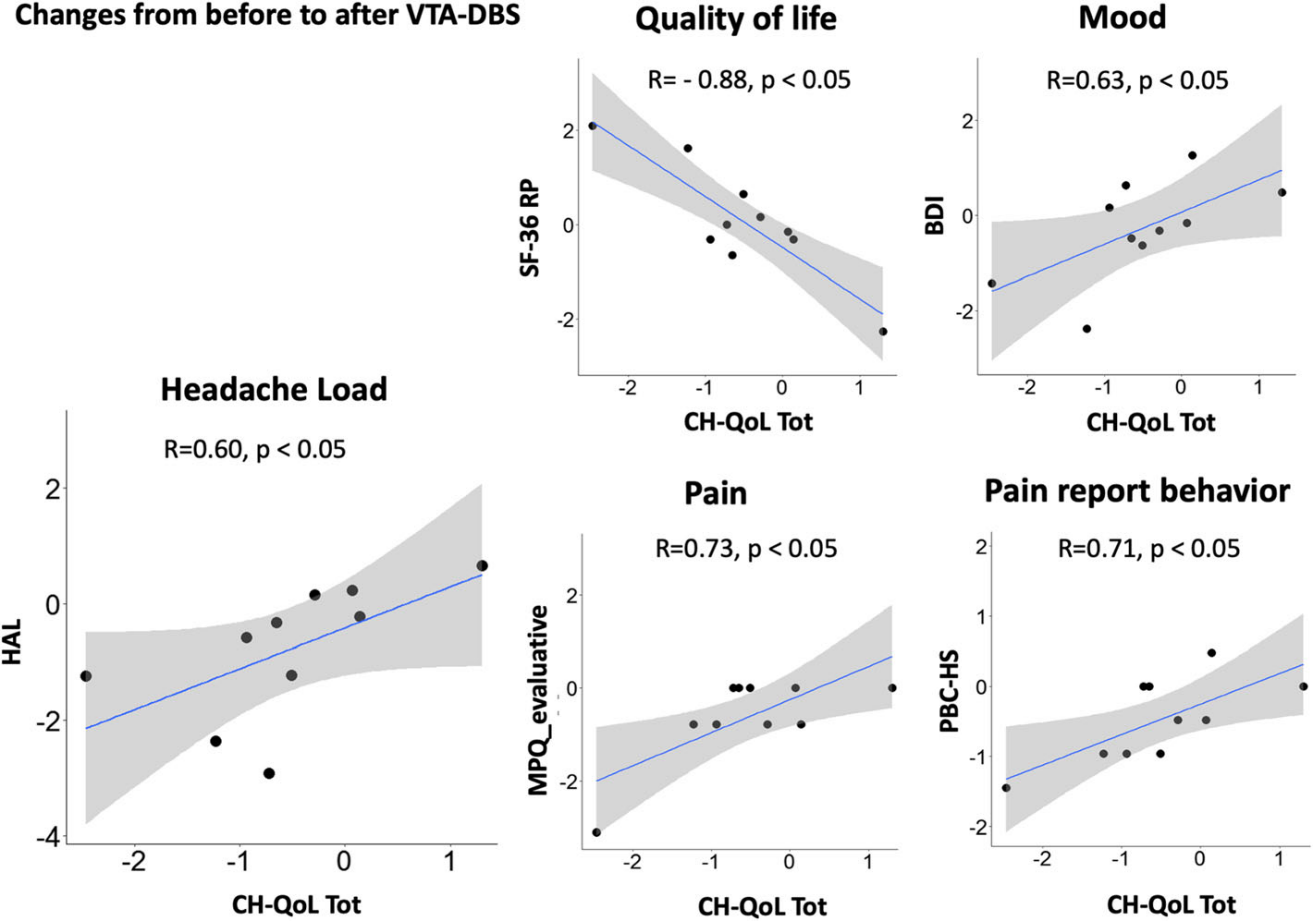
Scatter plots representing significant Pearson correlations between changes in CH-QoL total score and changes in other measures of quality of life (SF-36), mood (BDI-II) and pain (McGill Pain Questionnaire evaluative scale, HIT-6) and Pain Behaviour Checklist help-seeking behaviours (PBC-HS) from before to after VTA-DBS. Note: CH-QoL total higher scores indicate poorer health related quality of life. SF-36 higher scores indicate better health related quality of life, BDI-II, HIT-6, McGill Pain Questionnaire (MPQ) lower scores indicate better functioning. BDI-II Beck depression inventory-II, HAL headache load, HIT-6 headache impact test-6, RP role physical, Tot CH-QoL total score

Pearson correlational analyses were performed to explore the relationship between the change score in CH-QoL scale (before and after VTA-DBS) and the change scores in the clinical outcome composite score of headache frequency and severity (HAL) and the other measures of mood, pain and pain behaviour, and quality of life (see Fig. 2).

### Headache load

There was a significant negative correlation between CH-QoL total score and HAL *r* = -0.60, *p* = 0.03, indicating that lower scores on the CH-QoL (better QoL) are associated with the reduction of frequency, duration and severity of headache attacks suggesting the sensitivity to change of CH-QoL.

### Generic quality of life

We found a significant negative correlation between the CH-QoL total score and the SF-36 role physical *r* = -0.87, *p* < 0.05. This indicates that lower scores on the CH-QoL (better QoL) are associated with improvement of aspects of quality of life assessed by the generic SF-36 after VTA-DBS.

### Mood

We found significant positive correlations between the CH-QoL total score and ratings of mood on the BDI *r* = 0.63, *p* < 0.05, indicating that lower CH-QoL score (better QoL) is associated with a reduction of depressive symptoms after VTA-DBS.

### Pain

There were significant positive correlations between the CH-QoL total score and the evaluative domain on the McGill Pain Questionnaire *r* = 0.73, *p* < 0.05, and a correlation approaching significance with HIT6 *r* = 0.58, *p* = 0.06, indicating that lower/improved CH-QoL scores are associated with a reduction of pain evaluation and the impact of pain after VTA-DBS.

### Pain behaviour

We found significant positive correlations between the CH-QoL total score and the help-seeking domain on the Pain Behaviour Checklist (PBC) *r* = 0.71, *p* < 0.05, suggesting that lower/improved CH-QoL score is associated with reduction of pain related help-seeking behaviours after VTA-DBS.

## Discussion

Since there was no disease-specific measure of QoL for the most common TAC, cluster headache, we recently developed and validated the 28 item CH-QoL and demonstrated that it had good construct and convergent validity, internal consistency and test-retest reliability [6]. The aim of the present study was to evaluate the sensitivity of the CH-QoL scale to change by administering it to 10 patients with cluster headache before and one year or longer after VTA-DBS to determine whether it reflected the improvement in headache load observed following surgery. The results showed that the CH-QoL total score and the four subscales all reflected improved quality of life following VTA-DBS compared to before surgery, a change that was significant for the total score and the main subscale of ‘restrictions of ADL’. The sensitivity of the CH-QoL to change was further confirmed by two other aspects of the results. First, the change scores of the CH-QoL total score and the reduction of headache load were significantly related, indicating that the reduction of the frequency, duration and severity of headache attacks after VTA-DBS are reflected by the pre versus post-operative change scores of the CH-QoL. Second, the associations of change scores of the CH-QoL total score with change scores of the generic QoL measure the SF-36, indices of mood (BDI), pain (HIT-6 and McGill), and pain-related behaviors (Pain Behaviour Checklist-Help Seeking behaviours) were in the expected direction; all reflecting an association between the improvement of disease-specific and generic QoL, mood, pain and pain-related behaviours following VTA-DBS surgery.

HADS-A showed no significant association with the CH-QoL total score or with the “Pain and Anxiety” subscale. This might be because the CH-QoL subscale measures specific anxiety relating to having an attack rather than generalized anxiety symptoms as in the HADS-A. Also, having a newly implanted DBS device might have contributed to anxiety in many ways, such as fear of loss of effect, worries about maintenance and charging, concerns about infection in the period following surgery. While change on the ‘restrictions of ADL’ subscale was significant and the other three subscales of the CH-QoL also reflected improved functioning following VTA-DBS, these other features of CH-QoL such as ‘mood and interpersonal relations’ and ‘lack of vitality’ ‘pain and anxiety’ may require a longer time post-DBS to adequately and significantly reflect change following reduction of headache load, since interpersonal relations and vitality unlike daily activities may be aspects of quality of life that require a longer period for a move towards readjustment and ‘normalization’. This is a hypothesis thatcan be tested by further follow-up of this sample or other surgical or medically treated samples for a longer period of say 3 or 4 years after VTA-DBS.

As for most patients with cluster headache, standard medical treatment would entail medication, future studies could also further evaluate the sensitivity to change of CH-QoL by examining its responsiveness to change following effective medical treatment.

## Conclusions

In conclusion, the lack of a gold standard to assess QoL in CH is currently limited to using a combination of generic tests not explicitly devised to assess CH patients. Our study indicates that CH-QoL responds similarly to other validated generic scales, supporting CH-QoL’s validity and sensitivity to detect CH patients’ clinical changes following surgical treatment. Thus, these findings support the utility of CH-QoL for clinicians to assess responsiveness following other medical therapies. However, the small number of patients and the lack of testing for other medical therapies limit the generalizability of our results on CH-QoL’s sensitivity to change. Ultimately, confirmatory studies in a large population should determine whether CH-QoL could substitute the generic quality of life scales currently used in clinical practice.

## Abbreviations

ADL: activities of daily living
BDI-II: Beck depression inventory-II
CH: cluster headache
CH-QoL: cluster headache quality of life scale
HADS: Hospital anxiety and depression rating scale
HAL: headache load
HIT-6: headache impact test-6
QoL: quality of life
TACs: trigeminal autonomic cephalalgias
VTA-DBS: ventral tegmental area deep brain stimulation
